# Fused Deposition Modeling (FDM) 3D Printed Tablets for Intragastric Floating Delivery of Domperidone

**DOI:** 10.1038/s41598-017-03097-x

**Published:** 2017-06-06

**Authors:** Xuyu Chai, Hongyu Chai, Xiaoyu Wang, Jingjing Yang, Jin Li, Yan Zhao, Weimin Cai, Tao Tao, Xiaoqiang Xiang

**Affiliations:** 1 National Pharmaceutical Engineering Research Center, China State Institute of Pharmaceutical Industry, Shanghai, 201203 P.R. China; 2 Experiment Center for Science and Technology, Shanghai University of Traditional Chinese Medicine, Shanghai, 201203 P.R. China; 3 Department of Clinical Pharmacy, School of Pharmacy, Fudan University, Shanghai, 201203 P.R. China

## Abstract

The aim of this study was to explore the feasibility of fused deposition modeling (FDM) 3D printing to prepare intragastric floating sustained release (FSR) tablets. Domperidone (DOM), an insoluble weak base, was chosen as a model drug to investigate the potential of FSR in increasing its oral bioavailability and reducing its administration frequency. DOM was successfully loaded into hydroxypropyl cellulose (HPC) filaments using hot melt extrusion (HME). The filaments were then printed into hollow structured tablets through changing the shell numbers and the infill percentages. Physical characterization results indicated that the majority of DOM gradually turned into the amorphous form during the fabrication process. The optimized formulation (contain 10% DOM, with 2 shells and 0% infill) exhibited the sustained release characteristic and was able to float for about 10 h *in vitro*. Radiographic images showed that the BaSO_4_-labeled tablets were retained in the stomach of rabbits for more than 8 h. Furthermore, pharmacokinetic studies showed the relative bioavailability of the FSR tablets compared with reference commercial tablets was 222.49 ± 62.85%. All the results showed that FDM based 3D printing might be a promising way to fabricate hollow tablets for the purpose of intragastric floating drug delivery.

## Introduction

Oral drug administration is the predominant and most convenient route in clinical practice. However, oral drug absorption is often unsatisfactory and highly variable among the individuals due to the physiological variability such as irregular gastrointestinal transit and emptying time^1^. A short gastric residence time may result in incomplete drug release from the delivery vehicle, subsequently leading to poor therapeutic effect^2^. Several novel gastroretentive delivery systems have been explored to overcome the shortcomings above, including polymer bioadhesive systems, swelling and expanding systems, high-density systems and floating systems^3^. The floating systems are low-density systems that have sufficient buoyancy to float over the gastric contents and remain in the stomach for a prolonged and predictable period^4^. It can enhance the solubility of weakly basic drug in an acid environment and improve the patient compliance by decreasing dosing frequency^5^.

Domperidone (DOM) is a dopamine (D_2_) receptor antagonist, widely used in the treatment of gastroparesis and other conditions causing chronic nausea and vomiting^6^. It is a poorly water-soluble weak base (pKa_1_ = 7.8, pKa_2_ = 11.5) with good solubility in acidic condition whereas in neutral and alkaline environments its solubility is largely reduced^7^. The oral bioavailability of DOM was reported in the range of 13–17% probably as a result of both incomplete absorption and first-pass metabolism^6^. In humans, DOM is mainly absorbed in the upper gastrointestinal tract and peak plasma concentration was observed 30 min after oral (fasted) administration. It is recommended to take three times a day due to the short elimination half-life of about 7.5 h^6^. Therefore, DOM was considered to be a suitable candidate for intragastric floating sustained delivery to increase its oral bioavailability and reduce the frequency of administration^8, 9^.

The recent FDA approval of the first three dimensional (3D) printed tablets, Spritam (levetiracetam), opened a new chapter in 3D printing for pharmaceutical applications^10–12^. 3D printing is a layer-by-layer process to form 3D objects from digital designs using plastic and metal materials^13^. Amongst the different developed 3D printing technologies, fused deposition modeling (FDM) has several significant advantages such as low-cost manufacturing, the compatibility of using the combination of pharmaceutical grade polymers with APIs through hot-melt extrusion (HME) and the ability to fabricate hollow objects^14–16^. Previous pharmaceutical researchers attempted to use FDM-based 3D printing mainly in fabricating drug delivery systems with immediate or controlled release characteristics^17–19^, hot-melt extruding filaments of pharmaceutical grade polymers^15, 20, 21^, lowering printing temperatures^22^, and preparing personalized topical drug delivery together with 3D scanning^23^. To our knowledge, there is no report on the FDM based hollow tablets for intragastric floating sustained release (FSR) of drugs.

In FDM 3D printing, shells and infill are the key parameters which define the outline shape and inner support structure of an object. At least one shell is needed to print an object, and additional shells will add body’s strength and weight but consume more printing time and materials. Similarly, infill percentage is another parameter which could be adjusted from 0% to 100%, generating the object from completely hollow structure to fully solid filled structure. Based on this hollow structure concept, the aim of this study was to investigate the feasibility of printing DOM intragastric floating sustained release (DOM-FSR) tablets to reduce the frequency of administration to twice daily by changing the shell numbers and infill percentages. We also aimed to evaluate the *in vitro* and *in vivo* properties of these hollow structures.

## Results and Discussion

### Preparation of filaments and DOM-FSR tablets

**Figure 1:**
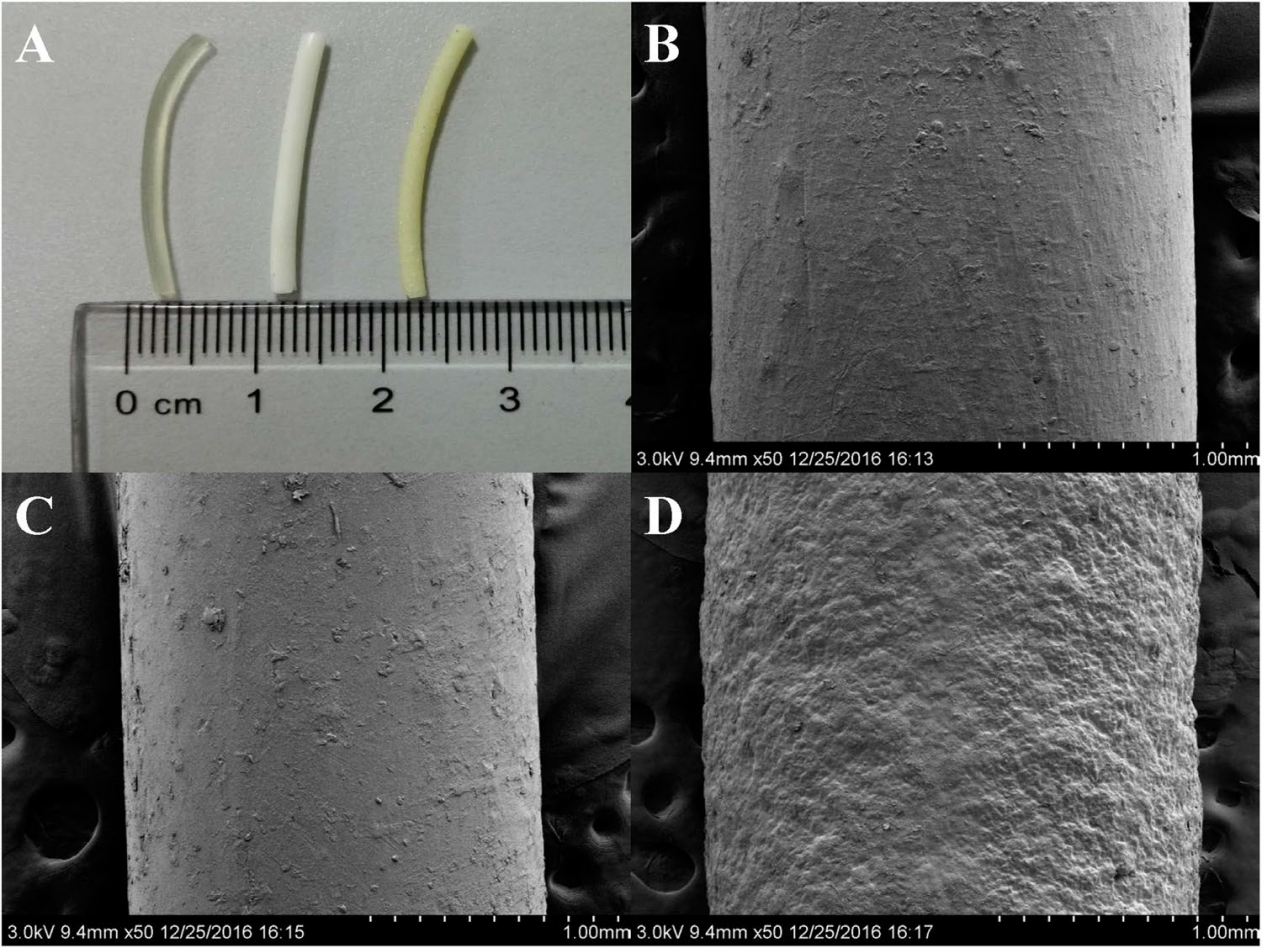
Image of HME fabricated filaments (**A**): HPC filament, DOM-HPC filament, and BaSO_4_-HPC filament (from left to right); SEM images of filaments: HPC filament (**B**), DOM-HPC filament (**C**), and BaSO_4_-HPC filament (**D**).

Polymer filaments are feed stock materials for FDM 3D printing. Several pharmaceutical grade polymers, including HPC, were recently investigated to obtain drug loaded filaments via HME^15, 17, 21^. A capsular device printed by FDM using HPC filament was explored for pulsatile drug release^16^. The rigid outer shell of HPC led to slow solvent penetration, and the inner loaded drug released immediately after a lag phase for about 2 h^16^. Enlightened by this paper, we developed the hollow-structured tablets for intragastric drug delivery. The outer shell was constructed by DOM-loaded HPC filaments, and the inner filled drug was replaced by air to maintain the whole tablets in a low-density state. The formulation and HME parameters of filaments are shown in Table S1. HPC, DOM/HPC (10/90, w/w), and BaSO_4_/DOM/HPC (10/10/80, w/w/w) were continuously extruded at a screw speed of 20–25 rpm. The hot-melt extrusion was performed in 145–150 °C and the torque was in the range of 10–20 N/cm without help of plasticizer. The extruded HPC filament has a transparent appearance while the HPC filaments loaded with DOM or BaSO_4_ have a white or slightly yellow opaque look (Fig. 1A), which indicated that the DOM and BaSO_4_ still existed in the form of particles after HME process.

**Figure 2:**
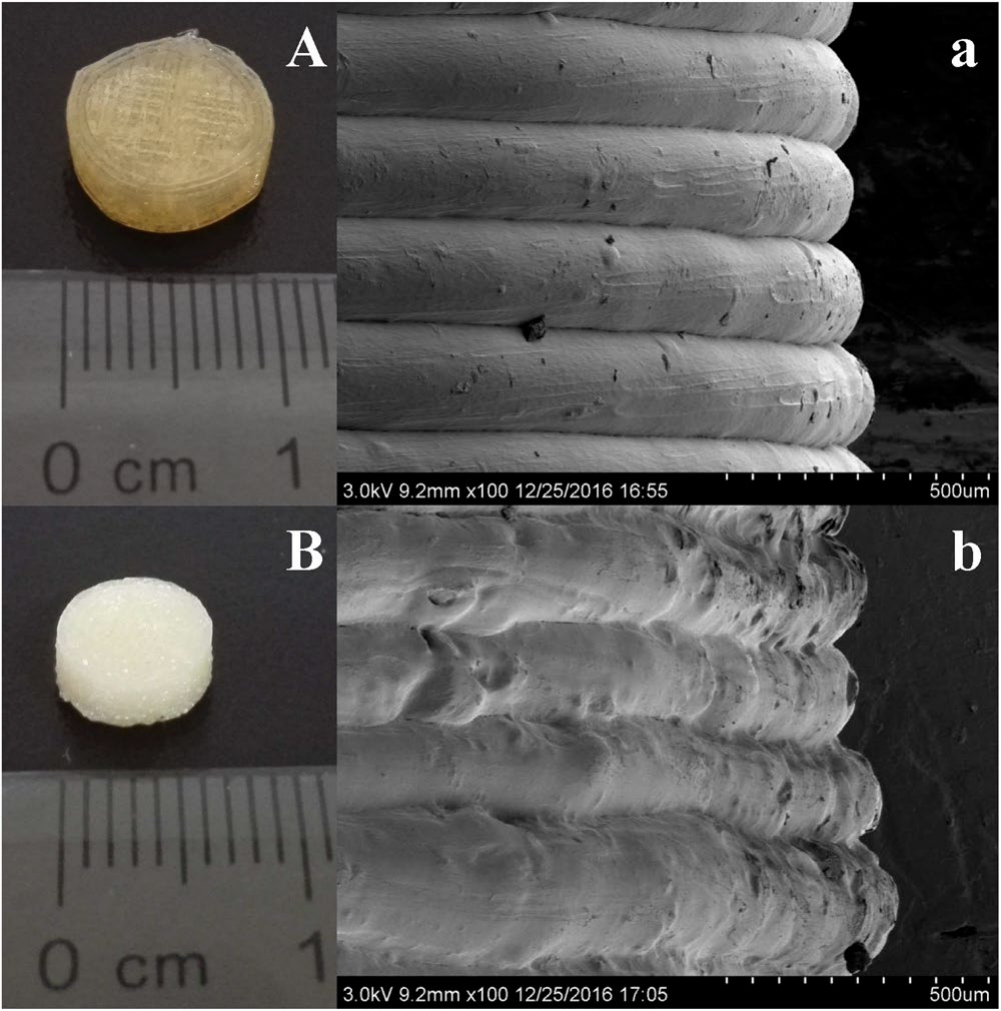
Images of 3D printed tablets: DOM-HPC tablet (**A**) and BaSO_4_-HPC tablet (**B**); SEM images of tablet side surfaces: DOM-HPC tablet (a) and BaSO_4_-HPC tablet (b).

Cylinder shaped tablets with different dimensions, shell numbers and infill percentages were printed using the filaments fabricated by HME. The formulation and designed physical parameter of DOM-FSR tablets was described in Table S2. The measured volume, weighted mass and calculated density were presented in Table S3. Tablets were designed and printed in two different sizes: the tablets with 10 × 3.2 mm (diameter × height, D × H) were used for the formulation research, while the tablets with 7.4 × 6 mm (diameter × height, D × H) were used for the *in vivo* evaluation in rabbits, considering the feasibility of feeding the tablets to rabbits in an integral state^24^. After FDM printing, tablets loaded with DOM had been converted from white (filament color) into transparent yellow (Fig. 2A), while BaSO_4_ labeled tablets remained white opaque look (Fig. 2B). This phenomenon indicated that the majority of DOM particles have turned into amorphous form with the incensement of preparation temperature.

### Characterization of filaments and DOM-FSR tablets

The use of high temperature during HME and FDM 3D printing can lead to the degradation of thermo-labile drugs and polymers^25^. In this work, no significant mass loss was found in the thermogravimetric analysis (TGA) from 145 °C to 150 °C (HME process temperature) for both pure materials and mixtures (Fig. S1). DOM is a thermal stable compound with a melting point about 249 °C, no significant mass loss was found from 150 °C to 210 °C, while only 0.4–0.6% of loss in mass was found in HPC and extruded filament samples. These results were further confirmed by the content determination (DOM were found to have recovery of 99.7–100.2% in both filaments and tablets samples). The differential scanning calorimetry (DSC) thermograms of micronized DOM, HPC, physical mixtures (HPC/DOM = 9:1), filaments (HPC/DOM = 9:1) and DOM-FSR tablets (HPC/DOM = 9:1) were shown in Fig. S2. The curve of micronized DOM showed an endothermic peak at approximately 249 °C. The endothermic peak of DOM was negative shift to approximately 229 °C in the case of the physical mixture, further shrunk and shifted to lower temperature in filaments, and completely disappeared after tablet printing. The percentages of crystallinity, were 83.7%, 22.8% and 0% for physical mixture, extruded filaments and printed tablets respectively, which indicated a gradually reduction through the thermal and mechanical related processes. FT-IR spectroscopy was conducted to confirm the interaction between DOM and HPC. As shown in Fig. S3, the characteristic peaks of DOM appeared in 1719 and 1694 cm^−1^ of C = O, 1489 cm^−1^ of C-N, and 732 cm^−1^ of C-Cl, while the spectrum of HPC showed the characteristic peaks at 3463 cm^−1^ of O-H, 2972 cm^−1^, 2933 cm^−1^, 1459 cm^−1^ and 1375 cm^−1^ of C-H, 1275 cm^−1^, 1120 cm^−1^ and 1080 cm^−1^ of C-O-C. The physical mixture, extruded filaments and printed tablets showed approximately the superimposition of the drug and HPC, except for a slight shifting of C = O to low wave numbers of 1718 and 1692 cm^−1^. This indicated hydrogen bonding existing between the double carbonyl group of DOM and the hydrogen group of HPC (Chemical structure of DOM and HPC were described in Fig. S4), which may account for the obvious low-temperature melting peak shift found in DSC spectra.

X-ray Powder Diffraction (XRPD) indicated typical crystalline peaks of pure DOM at 2Theta = 14.2, 15.7 and 22.8 (Fig. S3). These peaks were still detected in the physical mixtures, but decreased in filaments and disappeared in the tablets. The scanning electron microscopy (SEM) images (Fig. 1B,C,D) showed DOM-HPC filament had a similarly smooth surface like blank HPC filament, while BaSO_4_ loaded filament had a relatively rough surface due to the infusible BaSO_4_ of high melting point. Similarly, the lateral side of DOM-HPC tablet had a smoother layered view than BaSO_4_-HPC tablet (Fig. 2A,B). All these tendencies suggested that DOM crystal had transformed into partially crystallized form in filaments, and further into amorphous form with the increase of manufacturing temperature from 145–150 °C of HME to 210 °C of 3D printing.

### *In vitro* floating and release

**Figure 3:**
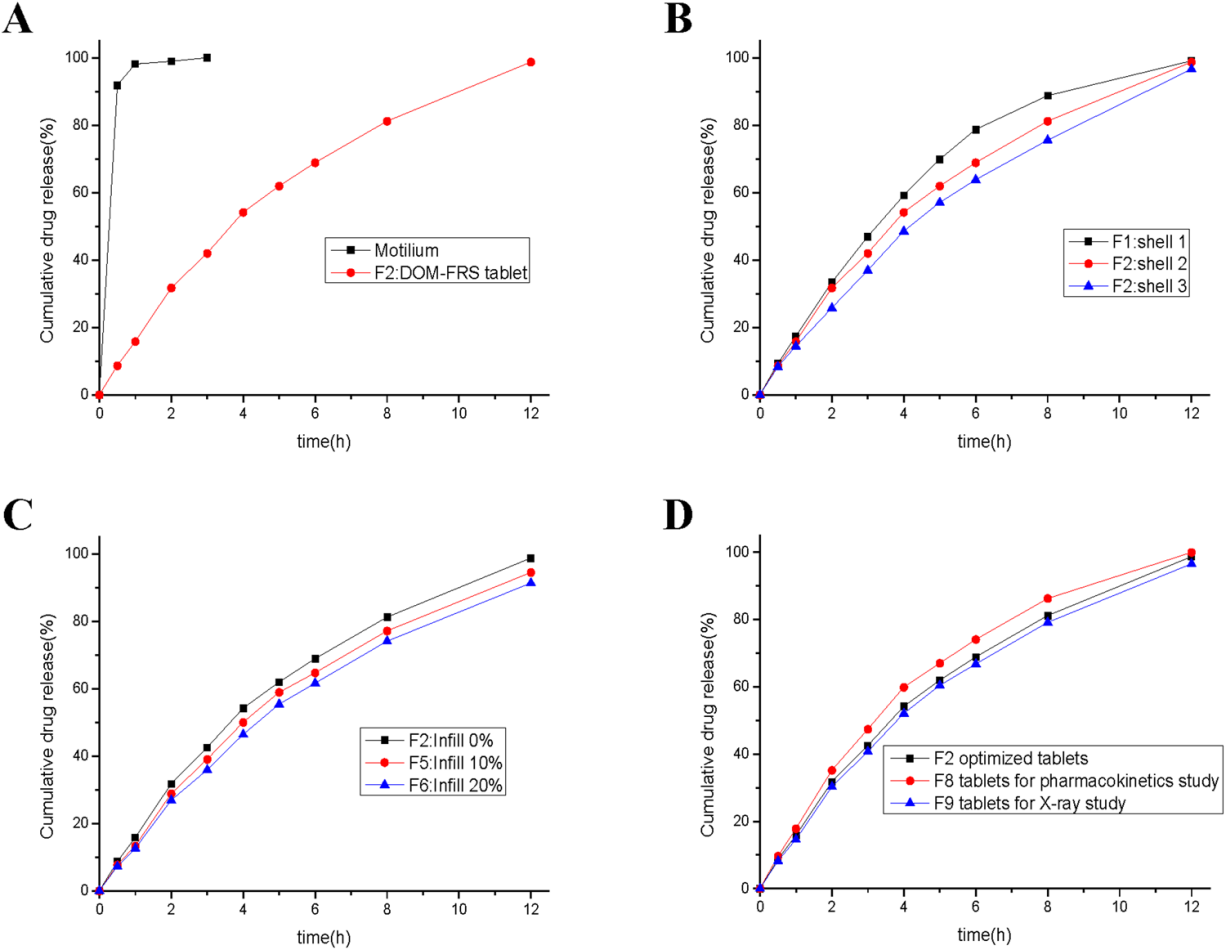
Drug release profiles of the formulations used in the experiments (n = 6): (**A**) comparison of commercial fast-release tablets and DOM-FSR tablets, (**B**) the influence of shell numbers, (**C**) the influence of infill percentages, and (**D**) comparison of the tablets with optimized parameters, tablets used for pharmacokinetic study, and tablets used for X-ray imaging.

In this experiment, the tablet densities were observed highly correlated with floating capacity. The optimized DOM-FSR tablets (Table S2, F2) with density of 0.77 g/cm^3^ had the capability of floating for more than 10 h (Fig. 3), while tablets with density of above 0.9 g/cm^3^ tended to sink to the bottom of the dissolution vessels in less than 1 h (Table S2). The optimized tablets (Table S2, F2) with 2 shells and 0% infill had a relative lower density of 0.77 g/cm^3^, and would be able to float for more than 10 h *in vitro* (Fig. 3). In contrast, the tablets with shells more than 3 or infill percentage more than 20% (Table S3, F4, F7), had a relative higher density of more than 0.9 g/cm^3^, would sink to the bottom of the dissolution vessels within less than 1 h. Considering the tablet’s density and buoyancy, the shell numbers and infill percentages were strictly limited (Table S2, S3).

**Figure 4:**
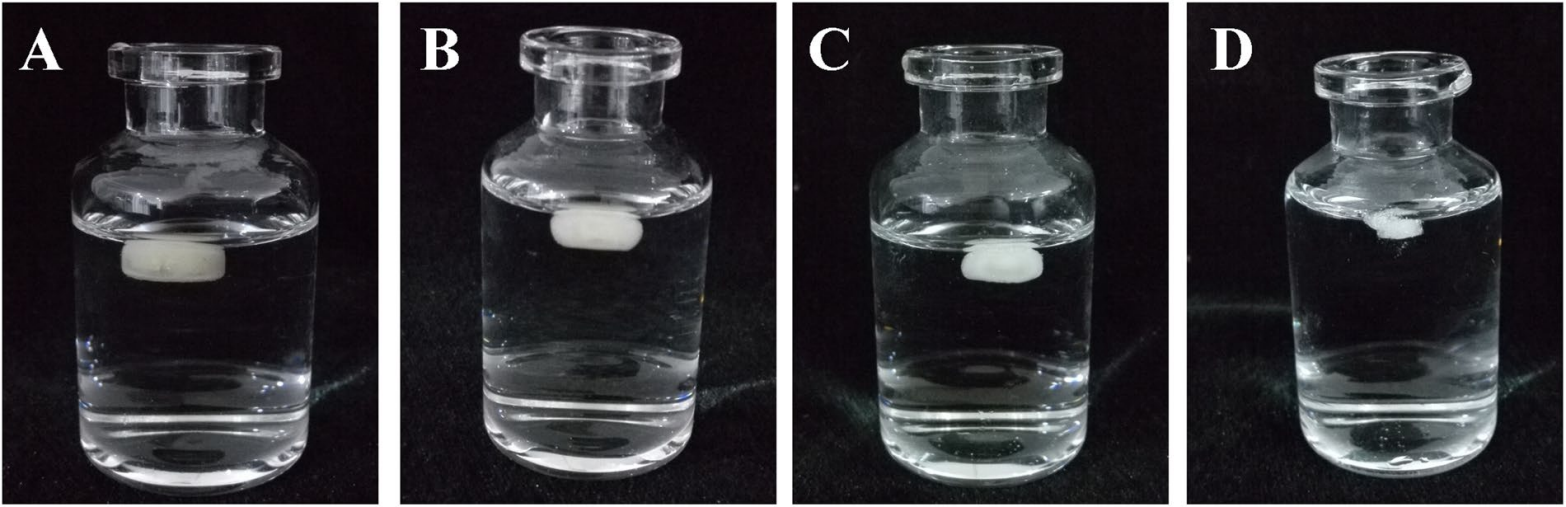
Photographs of DOM-FSR tablets floating in dissolution medium (0.1 N HCl solution) at 37 ± 0.5 °C. (**A**) t = 1 h, (**B**) t = 2 h, (**C**) t = 4 h and (**D**) t = 8 h.

An immediate drug release rate was achieved following the dissolution of two pills of Motilium^®^ (10 mg) in 0.1 N HCl, where 99.9% of the drug was released within 15 min, while the DOM-FSR tablet (Table S2, F2) released only 3% after 15 min, and achieved sustained release of 99.9% for 12 h (Fig. 4A). Different shell numbers (from 1–3, Table S2, F1–F3) and infill percentages (from 0–20%, Table S2, F2, F5 and F6) did not significantly influence the release rates of tablets (Fig. 4B,C). Although BaSO_4_ has a higher density of 4.5 g/cm^3^, 10% barium sulfate-loaded tablets showed a similar capability to float and sustain the drug release for more than 10 h *in vitro* (Table S2, Fig. 4D).

### *In vivo* floating and release

**Figure 5:**
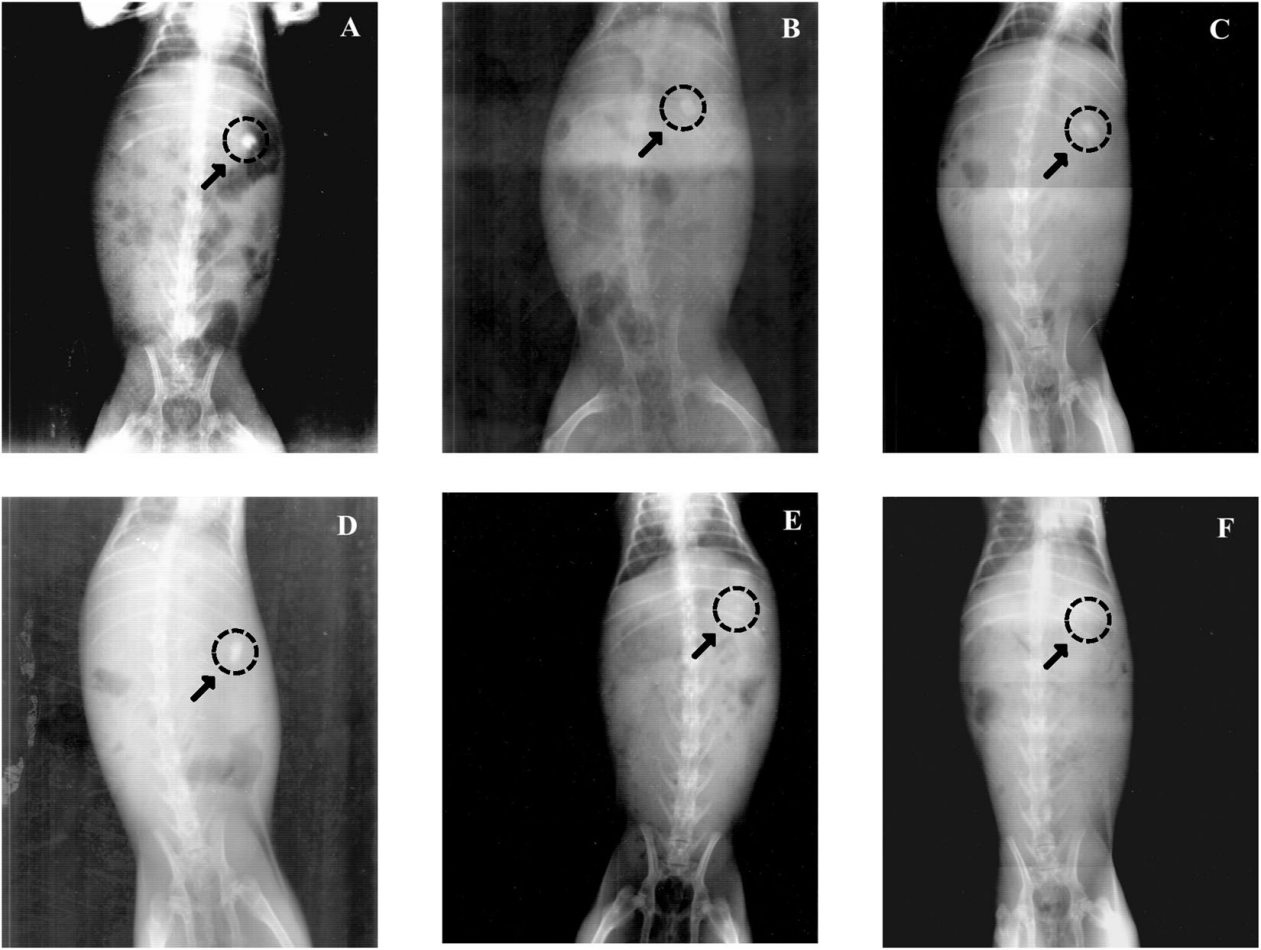
A-F X-rays indicating the positions of the BaSO_4_-labeled DOM-FSR tablets in the gastrointestinal tract of New Zealand rabbits at different time points. X-rays taken at (**A**) 2 h, (**B**) 4 h, (**C**) 6 h, (**D**) 8 h, (**E**) 10 h, and (**F**) 12 h.

**Figure 6:**
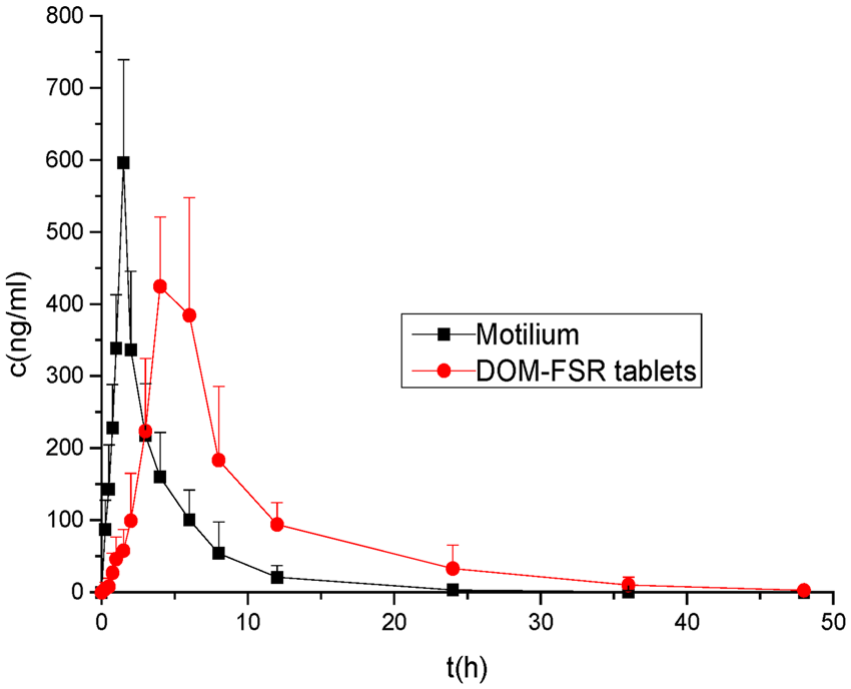
Plasma concentration-time (Means ± SD) profile of 20 mg DOM after oral administration of Motilium and DOM-FSR tablets in 8 New Zealand rabbits.

The rate and extent of oral drug absorption is determined by a complicated process, which is not only dependent on the drug physicochemical properties but also the interaction between the gastrointestinal tract and the administrated formulation^26^. The FDM based 3D printed tablets usually have a rigid structure like hard plastic with different features from conventional tablets^25^. To the best of the authors’ knowledge, this may be the first work to examine the *in vivo* behavior of FDM based tablets considering the interaction between gastrointestinal tract and tablets with unique physical characteristics. Figure 5 showed the X-ray images of the BaSO_4_-labeled tablets, which were floating in the New Zealand rabbits’ stomachs. The BaSO_4_-labeled tablets were clearly observed to stay in the stomach for 8 h (Fig. 5A–D), become fuzzy and indistinct at 10 h (Fig. 5E), and finally disappear at 12 h (Fig. 6F).

For pharmacokinetic studies, rabbits were administered with 20 mg DOM in commercial tablets (Motilium^®^, 2 pills) or in-house FSR tablets (Table S2, F8). The mean plasma concentration-time curve of the test and reference tablets in eight New Zealand rabbits were shown in Fig. 6 and the relevant pharmacokinetic parameters were summarized in Table 1. The relative bioavailability of the floating tablets was 222.49 ± 62.85% compared with the commercial tablets. The mean peak plasma concentration of DOM (C_max_) after a single oral dose of 20 mg was detected to be 596.167 ± 143.132 and 505.983 ± 43.175 ng/mL for the Motilium and DOM-FSR tablet, respectively. C_max_ was achieved after 1.5 h with the Motilium, while t_max_ of the DOM-FSR tablet was 5 h. These *in vivo* results indicated that the gastro floating tablets had exhibited a significantly prolonged steady release of the drug (p < 0.05) and was consistent with *in vitro* floating results. DOM is an insoluble weak base with pH-dependent release property, and its solubility was reported as low as 0.986 mg/L in water^27^. As described in the former research^7^, DOM powder could dissolve to 91% in 2 h in pH1.2 buffer, but only about 16% was achieved in up to 8 h in pH6.8 buffer. In that work^7^, an enteric hydroxypropyl methylcellulose phthalate (HPMCP) and insoluble ethylcellulose were used to prepare DOM coevaporates for the controlled release in gastrointestinal tract. One group was administrated with coevaporates containing 10 mg of DOM, and the other group was given conventional tablet with the same dose. The mean value of AUC_0→∞_ for coevaporate group was 247 h**·**ng/mL and for the conventional tablet group was 153 h**·**ng/mL. These results supported our work’s finding of the improvement of the oral bioavailability in rabbits with 3D printed FSR tablet. It can be inferred that the rapid dissolution of DOM in the acid medium of stomach might result in a quick precipitation after transferring into the neutral or basic environment of small intestine. Although DOM suffered from a first-pass metabolism after oral administration, the prolonged residence of DOM from 3D printed FSR tablet in the stomach might retard the formation of supersaturated solution and prevent precipitating in the small intestine. This might be the reasons explaining the incensement of oral bioavailability for DOM-FSR tablets.

**Table 1:** Pharmacokinetic parameters of Motilium and DOM-FSR tablets after oral administration of 20 mg (means ± SD, n = 8, *p < 0.05, **p < 0.01).

PK parameter	FSR tablets	Motilium
C_max_ (ng/mL)	505.98 ± 43.18	596.17 ± 143.13
t_max_ (h)	5.00 ± 1.10*	1.50 ± 0.10
t_1/2_ (h)	2.43 ± 0.74**	1.34 ± 0.43
AUC_0→t_ (h**·**ng/mL)	3310.59 ± 623.22**	1576.36 ± 504.39
AUC_0→∞_ (h**·**ng/mL)	3445.27 ± 682.25*	1861.99 ± 456.88
MRT_0→t_ (h)	7.75 ± 0.55**	3.16 ± 0.54
MRT_0→∞_ (h)	8.822 ± 0.82*	4.92 ± 2.13

## Conclusion

In summary, our study for the first time confirmed the feasibility of fabricating intragastric drug delivery device by FDM 3D printing, which holds high potential to develop a rapid and low-cost platform utilized for drug screening and personalized medical care. The DOM-FSR tablet with hollow structure was successfully fabricated and the buoyancy of tablets was closely related to their densities. Due to the rigid shells produced by melting deposition, HPC polymer chains dissociated slowly and thus produced the FSR affect. Physical characterization indicated that the state of DOM distributed in the HPC was converted into a solid dispersion with temperature elevating processes. Prolonged floating and release was observed both *in vitro* and *in vivo*. The increased oral bioavailability and steady plasma concentration of DOM demonstrated the promising application of DOM-FSR tablet to reduce the frequency of administration and improve patients’ compliance.

## Materials and Methods

### Materials

DOM (99.9%) purchased from Tianbin Biotechnology (Shaanxi, China), was jet milled (MC ONE, JETPHARMA SA, Switzerland) twice before the utilization in the preparation of the filament. The cumulative particle size distribution of milled DOM was D_10_ = 1.5 μm, D_50_ = 6.3 μm, and D_90_ = 14.7 μm measured by a laser diffractometer (Helos/Rodos, Sympatec GmbH, Germany). HPC (Klucel^TM^ EXF) was a gift from Ashland (Shanghai, China). Propranolol hydrochloride (internal standard, IS) was supplied by NIFDC (Beijing, China). All the other reagents were of either high performance liquid chromatography (HPLC) or analytical grade.

### Fabrication of filaments

Different ratios of DOM, BaSO_4_ and HPC were manually mixed together using a mortar and pestle before extruding in a twin-screw extruder (HAAKE MiniCTW, Thermo Scientific, Germany) equipped with a customized rod-shaped die (1.75 mm). The formulations and process parameters of filaments preparation were shown in Table S1. Only the filaments within the sizes of 1.75 ± 5 mm were further used for tablet printing.

### Tablet design and 3D printing

The cylinder shaped tablets with different infill percentages (0%, 10%, 20% and 30%) and shell numbers (1, 2, 3 and 4) were prepared to investigate the floating capability of the tablets (Tables S2 and S3). The templates were designed by AutoCAD^®^ 2014 (Autodesk Inc., USA) and fabricated with the previous HME prepared filaments using a desktop fused-deposition modeling 3D printer (Replicator 2X, MakerBot Inc., USA). The printing parameters were set as follows: extruder temperature: 210 °C; resolution: standard; platform temperature: 30 °C; travel speed: 90 mm/s; height of the layer: 0.2 mm; thickness of roof and floor: 0.8 mm; infill pattern: linear. Raft and Scotch Blue painter’s tape 50 mm (3 M, Shanghai, China) were applied to improve the adhesion of printing tablets to the building platform and removed after printing. The tablet height (H) and diameter (D) were measured by vernier caliper. The tablet weight (W) and the circular constant (π) were also used to calculate the tablet density (ρ) by the following equation (1). The formulation and physical parameters of DOM-FSR tablets were shown in Tables S2 and S3.

1 $$\rho =\frac{W}{{(\frac{D}{2})}^{2}\times \pi \times H}$$

### Characterization of filaments and DOM-FSR tablets

The images of the filaments and floating tablets were obtained using a Sony NEX-5 camera equipped with a macro lens. The surface morphologies of samples were photographed with a Hitachi S-4800 SEM. DSC and TGA were used to characterize the thermo properties under pure nitrogen atmosphere through all the analysis. For DSC analysis, the sample of 8–10 mg was placed in an alumina pan with a lid and measured by a TA Q2000 DSC analyzer at a heating rate of 10 °C/min. The percentage of crystallinity (POC) was calculated according to the equation (2)^28^. For TGA analysis, approximately 5 mg of samples were heated at 10 °C/min in an open aluminum pans using a TA Q500 TGA. FT-IR spectra were applied to record the interaction of DOM and HPC using a Nicolet IS10 Fourier transform infrared spectrometer (Thermo Electron Scientific Instruments LLC, USA) with a frequency range from 4000–650 cm^−1^. A Power X-ray diffractometer, Bruker D8 ADVANCE phaser with Lynxeye was applied to assess the physical form at a scanning rate of 6°/min in the 2θ range from 10° to 90°.

2 $${\rm{POC}}( \% )=\frac{{{\rm{\Delta }}{\rm{H}}}_{{\rm{s}}}}{{{\rm{\Delta }}{\rm{H}}}_{{\rm{DOM}}}\times {\rm{W}}\,}\times 100 \% $$

where ΔH_s_ and ΔH_DOM_ are the melting enthalpy of the test sample and pure DOM, respectively. W is the weight fraction of DOM in the samples.

### Sample analysis by HPLC-UV

To determinate the drug content, a tablet or a filament section (approx.0.1 g) was cut into small pieces and placed in a 50 mL volumetric flask containing methanol. The flask was ultrasounded for 30 min to ensure the complete dissolution of DOM. The dissolved sample was then filtered through 0.22 μm nylon filters (Anpel, China) and the concentration of DOM was determined by HPLC-UV system (LC-20AD prominence series HPLC system, Shimadzu, Japan) with Labsolutions software. The validated HPLC analysis entailed injecting 20 μL of samples using mobile phase consisted of methanol (60%) and 0.5% ammonium acetate solution (40%), through a Thermo 5μm C_18_ column (150 × 4.6 mm, Thermo, UK), maintained at 35 °C. The flow rate of mobile phase was 1.0 mL/min and the detection was performed at a wavelength of 285 nm. All the measurements were carried out in duplicate.

### *In vitro* dissolution and floating study

The *in vitro* release rate of DOM from DOM-FSR tablets (n=6) was evaluated using a USP dissolution apparatus II machine (RC806D, TDTF, China). The Dom-FSR tablets were placed in 900 mL of 0.1 N HCl release medium at 37 ± 0.5 °C and agitated at 50 rpm. At the predetermined intervals, samples were drawn and analyzed using a HPLC-UV method with the same chromatographic conditions as the content assay. To take the floating image clearly, DOM-FSR tablets were taken from dissolution vessels at different times, placed in 15 mL vials with dissolution media, and taken photos immediately.

### *In vivo* dissolution and floating study

#### Protocol

The pharmacokinetics and X-ray imaging experiments were approved by the Medical Animal Ethics Committee of Shanghai University of Traditional Chinese Medicine in accordance to the National Institute of Health Guide for the Care and Use of Laboratory Animals. A total of 12 male 4-month-old New Zealand White rabbits weighting 1.7–2.5 kg were purchased from SLAC Co., Ltd. (Shanghai, China) and acclimated for at least one week before use. All rabbits were housed in individual cages under controlled temperature of 21–23 °C with a regular 12 h of light-dark schedule. Food and tap water were provided ad libitum.

A single-dose two-period crossover pharmacokinetic study was conducted in eight healthy male New Zealand rabbits with a one-week washout period. After overnight fasting, a little low-calorie food was given before the experiment. The rabbits, weighing 1.8–2.3 kg, were randomly divided into two groups. One group was given the Dom-FSR tablets (Table S2, F8) containing 20 mg DOM and the other group was given two reference commercial DOM tablets (Motilium^®^, Xian-Janssen, China) with the same dose of DOM using a pet piller. The rabbits were given food 8 h after the administration of the tablets, but they had free access to water all the time. For the test and reference groups, blood samples were collected from the ear vein and placed in heparinized plastic centrifuge tubes immediately before and then 0.25, 0.5, 0.75, 1, 1.5, 2, 3, 4, 6, 8, 12, 24 and 48 h after dosing. These samples were processed by centrifugation for 10 min at 4500 rpm. The plasma samples were stored at −20 °C until analysis.

For radiographic studies, 10% (w/w) barium sulfate was incorporated to make the DOM-FSR tablets opaque, considering the balance of buoyancy and X-ray image view effect^29^. After overnight fasting, three healthy New Zealand rabbits weighing 2.0–2.5 kg were fed with a small amount of low-calorie food and water. One hour later, one barium sulfate-labeled tablet (Table S2, F9) was given to every animal using a pet piller. After administration, the rabbits were fed with 15 mL of water. At different times (2, 4, 6, 8, 10 and 12 h), the animals were kept in a standing position with a fixed distance from the X-ray source and radiographs were obtained with constant parameters. The rabbits were given 1.5 mL water every hour and had access to food 8 h after the tablet administration.

### Plasma sample analysis by HPLC-FLD

A liquid-liquid extraction process and HPLC fluorometric detection method moderately optimized from literatures^30, 31^ were employed to analyze the plasma samples. 200 μL plasma was mixed with 20 μL IS solution (1.024 μg/mL propranolol hydrochloride methanol solution) and 100 μL 0.1 mol/L NaOH solution. After 1 min of vortex-mixing, 3 mL CH_2_Cl_2_ was added being followed by vortex-mixing for 5 min. After centrifugation at 4500 rpm for 5 min, the organic layer was transferred to a new tube to separate with debris. After the first time of extraction, another 3 mL CH_2_Cl_2_ was added and repeated extraction was made to increase the recovery of DOM. The combined organic layer was evaporated to dry under pure nitrogen at 40 °C. The residue was reconstituted with 200 μL of the mobile phase and vortexed for 10 min. After centrifugation for 5 min at 10000 rpm, a 50 μL of aliquot was injected into the HPLC system for analysis.

The HPLC analysis was performed on a Shimadzu LC-20AD prominence series liquid chromatography system equipped with a RF-10A spectrofluorometiric detector (Shimadzu, Kyoto, Japan). The detection wavelength was set at 282 nm for excitation and 328 nm for emission. The column was Kromasil 100-5 C_18_ (4.6 × 250 mm, i.d., 5 μm; AKZO NOBEL, Sweden) and maintained at 40 °C by a CTO-20AC column oven. A guard column Athena C_18_ (4.0 × 20 mm, i.d., 5 μm, CNW, Shanghai, China) was attached before the analytical column. The mobile phase was 0.02 M phosphate buffer (pH3.5)-acetonitrile (25:75, v/v) with a flow rate of 1 mL/min. The developed method was validated before the samples analysis. The linearity was obtained in the range of 10–1000 ng/mL with an r (correlation coefficient) value of 0.999 and the LLOQ was 1 ng/mL.

### Pharmacokinetic data analysis

Data analysis of pharmacokinetic parameters was carried out using the software DAS 2.1.1. (Mathematical Pharmacology Professional Committee of China, Shanghai, China). Non-compartmental model was applied to calculate the pharmacokinetic parameters. The student’s *t*-test was performed to determine the significance of difference between the pharmacokinetic parameters. The level of significance was defined as *p* value < 0.05 using the statistical software SPSS (version 22.0).

## Electronic supplementary material

Supplementary information
