# Supplementary material for: Fused Deposition Modeling (FDM) 3D Printed Tablets for Intragastric Floating Delivery of Domperidone
Source: Sci Rep. 2017 Jun 6;7:2829. doi: 10.1038/s41598-017-03097-x (PMC5460192; doi:10.1038/s41598-017-03097-x)
Supplement: Supplementary file 1 — Supplementary information [file 41598_2017_3097_MOESM1_ESM.pdf]

Supplementary information for

Fused Deposition Modeling (FDM) 3D Printed Tablets for Intragastric Floating Delivery of Domperidone

Xuyu Chai[1], Hongyu Chai[1], Xiaoyu Wang[2], Jingjing Yang[1], Jin Li[1], Yan Zhao[1], Weimin Cai[3], TaoTao[1]&Xiaoqiang Xiang[3]

[1]National Pharmaceutical Engineering Research Center, China State Institute of Pharmaceutical Industry, Shanghai, 201203, P.R. China

[2]Experiment Center for Science and Technology, Shanghai University of Traditional Chinese Medicine, Shanghai, 201203, P.R. China

[3]Department of Clinical Pharmacy, School of Pharmacy, Fudan University, Shanghai, 201203, P.R. China

Correspondence and requests for materials should be addressed to T.T. (email: taotao@sinopharm.com) or X.X. (email: xiangxq@fudan.edu.cn)

Table S1. Formulation and extrusion parameters of filaments

Formulation(weight ratio)	Extrusion temperature (°C)	Screw speed(rpm)	Torque(N/cm)
HPC-EXF	145	20-25	10-18
HPC-EXF:DOM=90:10	145	20-25	10-20
HPC-EXF:DOM:BaSO4= 80:10:10	150	20-25	12-20

Table S2. Formulation and designed physical parameters of DOM-FSR tablets

Formulation code	Formulation composition (weight ratio) (%)	Designed dimensions (D×H) (mm)	Shell number	Infill percentage (%)
F1	HPC-EXF:DOM(90:10)	10×3.2	1	0
F2	HPC-EXF:DOM(90:10)	10×3.2	2	0
F3	HPC-EXF:DOM(90:10)	10×3.2	3	0
F4	HPC-EXF:DOM(90:10)	10×3.2	4	0
F5	HPC-EXF:DOM(90:10)	10×3.2	2	10
F6	HPC-EXF:DOM(90:10)	10×3.2	2	20
F7	HPC-EXF:DOM(90:10)	10×3.2	2	30
F8	HPC-EXF:DOM(90:10)	7.4×6	2	0
F9	HPC-EXF:DOM:BaSO4(80:10:10)	7.4×6	2	0

Table S3. Measured physical parameters and floating time duration of DOM-FSR tablets (n=6)

Formulation code	Measured volume (mm^3)	Measured mass (mg)	Tablet density (mg/mm^3)	Floating time (h)
F1	258.97±3.30	181.02±0.95	0.70±0.01	>8
F2	261.95±4.71	200.67±0.41	0.77±0.01	>10
F3	260.18±3.95	219.57±5.43	0.84±0.02	>10
F4	257.92±2.70	239.05±3.34	0.93±0.03	<1
F5	260.70±2.50	217.42±1.84	0.83±0.01	>10
F6	261.48±4.47	231.34±0.91	0.88±0.01	>10
F7	260.12±6.15	244.63±1.73	0.94±0.02	<1
F8	261.22±2.18	199.85±2.09	0.77±0.01	>10
F9	260.98±2.47	225.45±0.40	0.86±0.01	>10

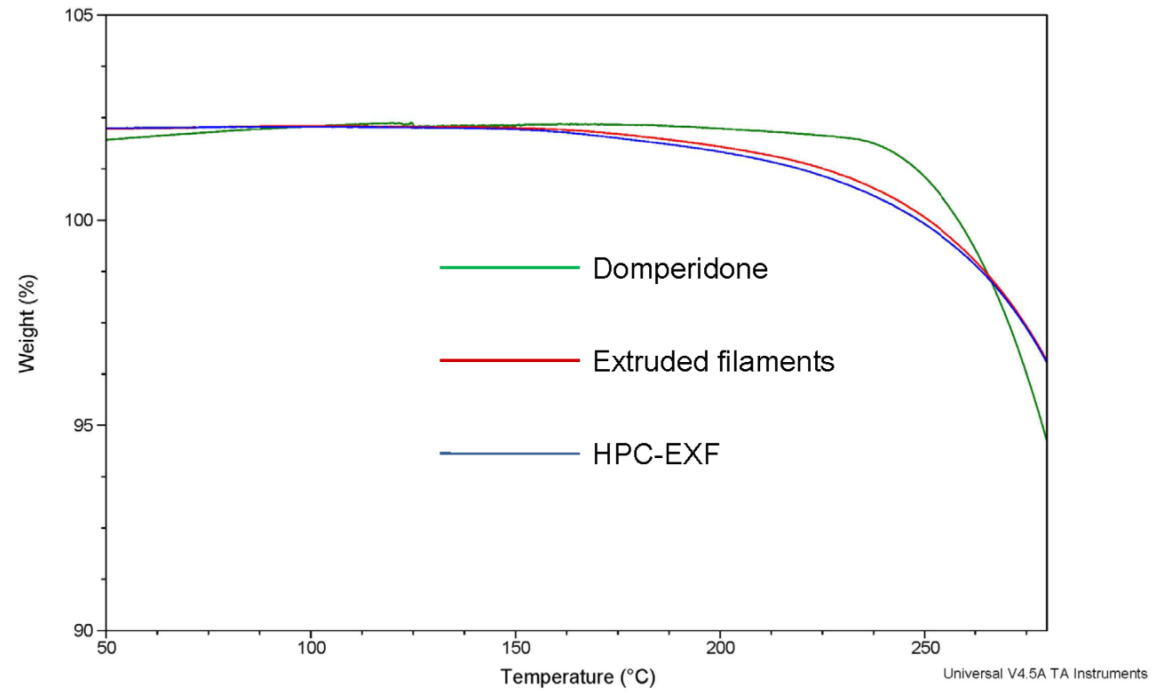

Fig.S1.TGA thermo traces for DOM, HPC-EXF and extruded filaments (DOM10%).

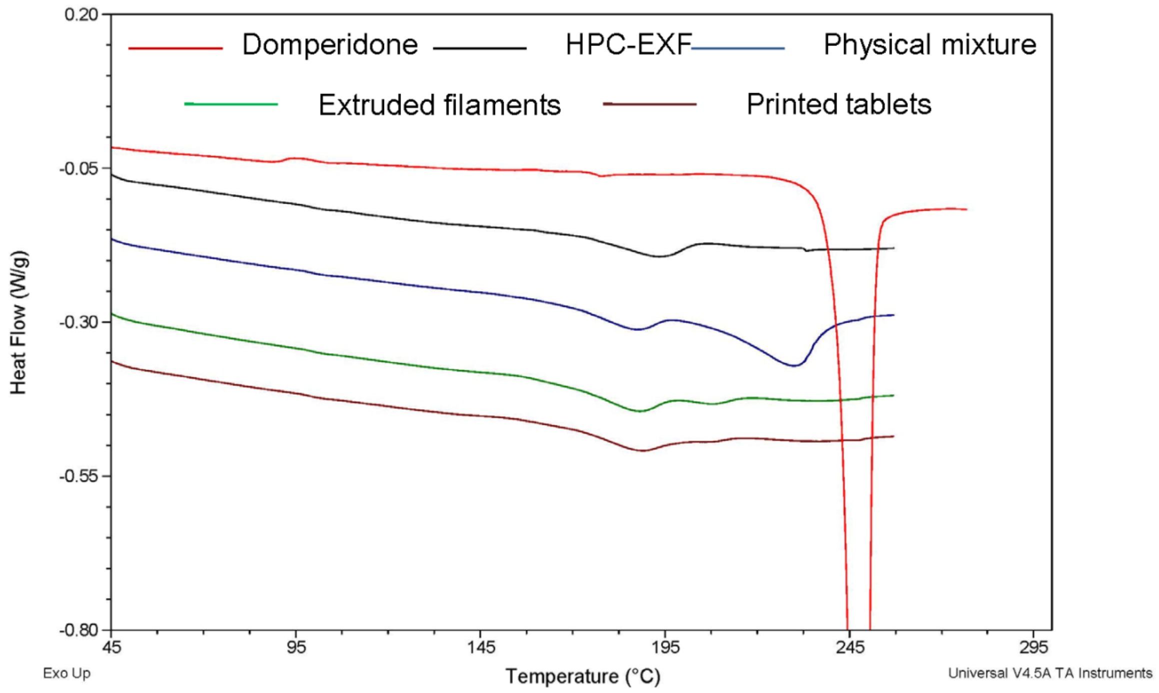

Fig.S2. DSC thermographs of DOM, HPC-EXF, physical mixture (DOM10%), extruded filaments (DOM10%) and printed tablets (DOM10%).

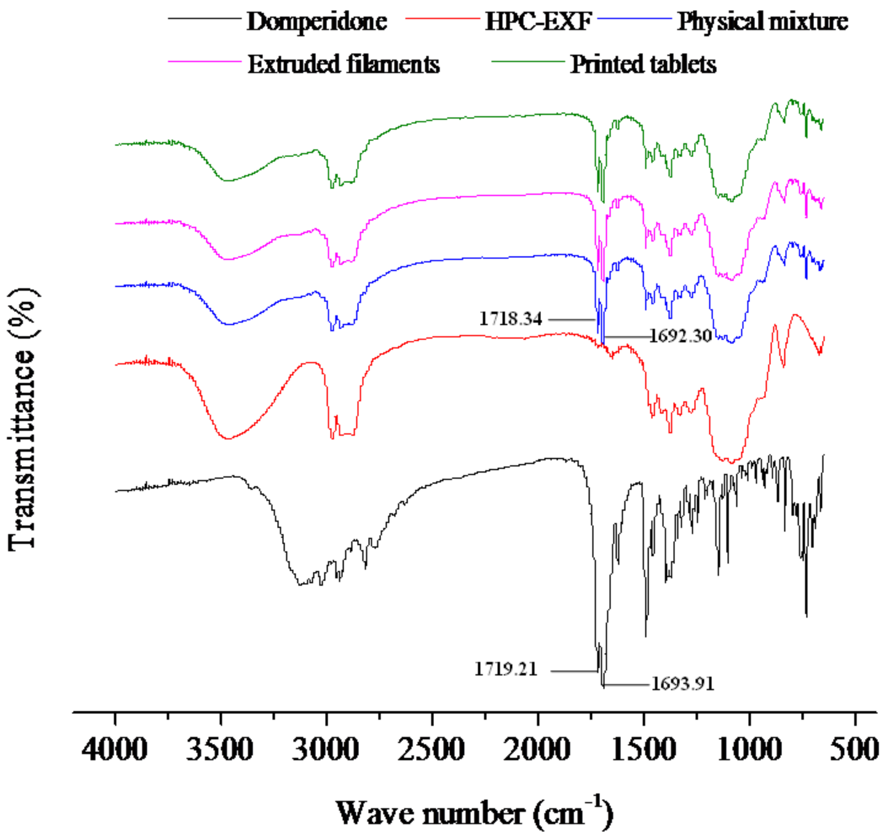

Fig.S3. FT-IR spectra of DOM, HPC-EXF, physical mixture (DOM10%), extruded filaments (DOM10%) and printed tablets (DOM10%).

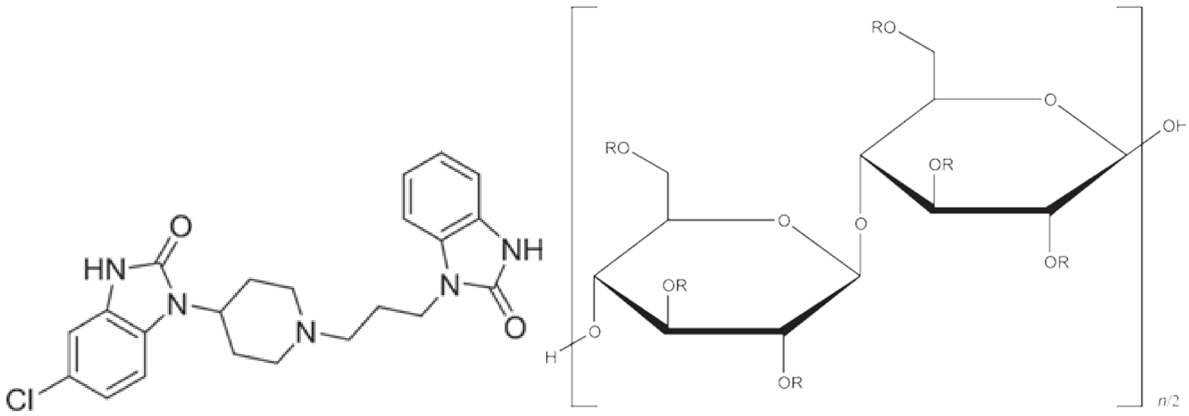

Fig.S4. Chemical structure of DOM and HPC (R is H or $[CH_2CH\ (CH_3)\ O]_m H$ where m is a common integral number of cellulose derivatives).

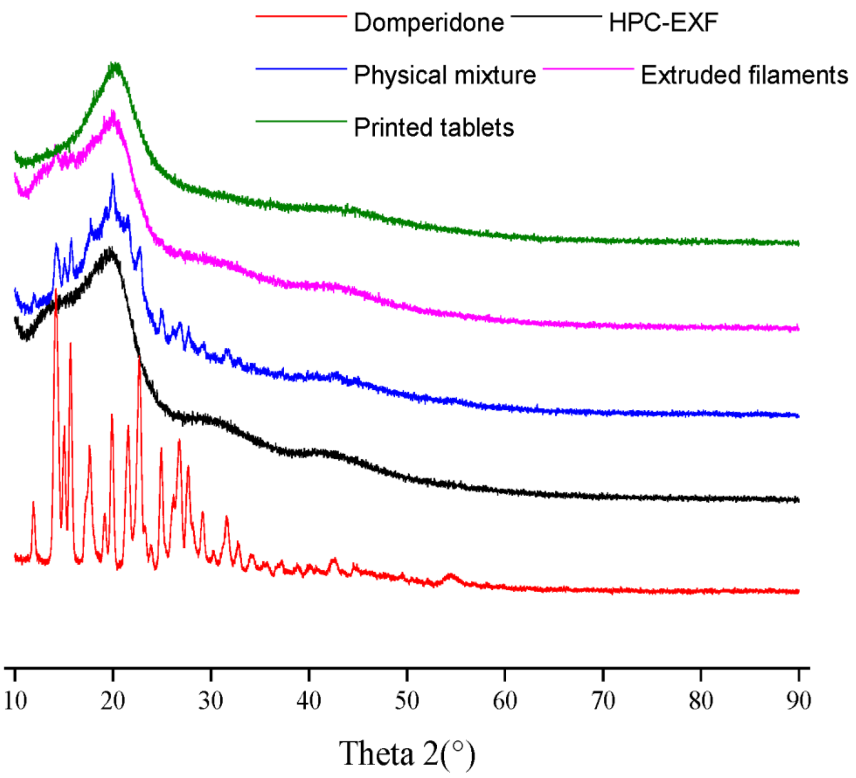

1

Fig.S5. XRPD spectra of DOM, HPC-EXF, physical mixture (DOM10%), extruded filaments (DOM10%) and printed tablets (DOM10%).
